# CAN ARTIFICIAL INTELLIGENCE HELP ADDRESS THE 5MS OF GERIATRICS?

**DOI:** 10.1093/geroni/igae098.0437

**Published:** 2024-12-31

**Authors:** Christopher Kaufmann

**Affiliations:** University of Florida College of Medicine, Gainesville, Florida, United States

## Abstract

Over the past year, the world has witnessed an explosion in the availability of tools employing artificial intelligence (AI) to do things never imagined possible. In parallel to its worldwide growth, applications of AI have also entered the healthcare sector. Specifically, AI tools have the potential to assist healthcare professionals in making judicial clinical decisions and to empower patients to monitor their health and make informed healthcare choices. Still, the application of AI to the geriatrics and gerontology field is limitless and represents a major opportunity to promote successful aging and independence of older persons. The 5Ms of Geriatrics is a framework used in clinical practice for assessing an older adult patient’s cognitive and physical functioning. The key Ms include Mind, Mobility, Medications, Multicomplexity, and what Matters Most, and addressing these five domains is essential clinical practice for improving health outcomes, particularly for older patients. In this presentation, we will explore the current applications and approaches of using AI to specifically address each of the 5Ms. The discussion will focus on the applicability of AI to address each domain and provide key examples of how AI, to date, has addressed each domain. The presentation will also propose key opportunities for future applications of AI in the geriatrics and gerontology field, with the ultimate goal of better providing for the healthcare of older people and promoting aging in place and successful aging.

